# Cardiometabolic index and the risk of new-onset chronic diseases: results of a national prospective longitudinal study

**DOI:** 10.3389/fendo.2024.1446276

**Published:** 2024-10-21

**Authors:** Liyuan Zhuo, Mingxi Lai, Lulu Wan, Xuan Zhang, Ronglin Chen

**Affiliations:** ^1^ Department of Intensive Care Unit, Longgang Central Hospital of Shenzhen, Shenzhen, Guangdong, China; ^2^ Department of Internal Medicine, Shenzhen Baoan Maternal and Child Health Hospital, Shenzhen, Guangdong, China; ^3^ Shantou University Medical College, Shantou, Guangdong, China

**Keywords:** CMI, chronic diseases, longitudinal study, CHARLS, new-onset

## Abstract

**Background:**

The cardiometabolic index (CMI) has emerged as a novel marker for evaluating the distribution and dysfunction of visceral adipose tissue, yet its correlation with numerous diseases, particularly new-onset chronic conditions, remains underexplored. Therefore, we aim to explore the association of cardiometabolic index (CMI) and new-onset chronic diseases.

**Methods:**

The analysis utilized data from the China Health and Retirement Longitudinal Study, with a baseline in 2011 and follow-ups biennially until 2020. Fourteen new-onset chronic diseases were diagnosed based on self-report, and separate cohorts were created for each disease. CMI was calculated as triglycerides/high-density lipoprotein cholesterol multiplied by the waist-to-height ratio. Cox proportional hazards models were used to assess the association between CMI and new-onset chronic diseases, while restricted cubic spline (RCS) models were employed to explore potential nonlinear effects. Additional and sensitivity analyses included Kaplan-Meier survival curves, subgroup analyses, multiple imputations, and exclude outcome events at the first follow-up.

**Results:**

Higher levels of CMI were associated with an increased risk of new-onset hypertension (HR=1.05, 95% CI=1.04-1.06, *P*<0.001), diabetes (HR=1.08, 95% CI=1.06-1.09, *P*<0.001), dyslipidemia (HR=1.07, 95% CI=1.06-1.09, *P*<0.001), liver disease (HR=1.05, 95% CI=1.03-1.07, *P*<0.003), and stroke (HR=1.04, 95% CI=1.02-1.06, *P*<0.001), although the association with stroke was not significant after adjusting for confounders (HR=1.02, 95% CI=1.00-1.05, *P*=0.054). Participants in the highest quartile of CMI had a significantly higher risk of these diseases compared to those in the lowest quartile. RCS analyses showed a significant nonlinear relationship between CMI and the risk of these diseases above.

**Conclusions:**

CMI showed a significant positive association with the risk of new-onset chronic diseases such as hypertension, diabetes, dyslipidemia, and liver disease. Future applications of CMI hold promise as an effective marker for early identification of chronic disease risk.

## 1 Background

Chronic diseases place a huge and growing burden on individuals, families and society, and such patients often suffer from multiple chronic diseases that require more complex and specialized healthcare services and higher healthcare costs, particularly among the elderly population (1, 2). China, as the country with the largest elderly population globally, is projected to have 400 million people over the age of 65 by 2050, thereby facing substantial challenges related to aging (3). An epidemiological study found that over 75.8% of Chinese residents aged 60 and above suffer from at least one chronic disease (4). Chronic diseases have become a major challenge to global health, with the negative consequences of ill health, disability and death creating an increasing economic burden, especially in low-and middle-income countries (5, 6).

The cardiometabolic index (CMI), calculated as triglycerides/high-density lipoprotein cholesterol (TG/HDL-C) multiplied by the waist-to-height ratio (WHtR), has emerged as a new marker for assessing the distribution and dysfunction of visceral adipose tissue, as well as the risk of obesity-related metabolic diseases (7–9). In a Chinese community-based study, Shi et al. found that CMI could serve as a valid and cost-effective indicator for screening and quantifying diabetes among Chinese individuals (10). Furthermore, CMI is considered to be strongly associated with hypertension, cardiovascular disease, metabolic associated fatty liver disease (MAFLD), and kidney disease, underscoring its potential as an indicator of metabolism-related diseases (11–13).

However, the association of CMI with various other diseases, particularly with new-onset chronic diseases, remains underexplored. Most previous studies on CMI and chronic diseases are based on cross-sectional data and require validation through large-scale longitudinal studies (12, 14, 15). The coexistence of various chronic diseases may also have an impact on each other, especially in cardiovascular diseases (16, 17). Meanwhile, given the high prevalence of chronic diseases and their adverse consequences among older adults, understanding the association between the CMI and new-onset chronic diseases is crucial.

To address this gap, we utilized data from the China Health and Retirement Longitudinal Study (CHARLS) to explore the longitudinal associations between CMI and 14 new-onset chronic diseases (including hypertension, diabetes, dyslipidemia, cancer, lung disease, liver disease, heart disease, stroke, kidney disease, digestive disease, arthritis or rheumatism, asthma, memory disease, and psychiatric disease), with the aim of providing a scientific and objective references in this area to address health issues related to aging, etiology, and the early intervention and prevention of chronic diseases.

## 2 Methods

### 2.1 Data sources

CHARLS, initiated in 2011, is a comprehensive survey aimed at Chinese residents aged 45 and older. It utilizes a complex, multistage probability sampling design to ensure national representativeness. The survey covers 450 villages and communities across 28 provinces, including autonomous regions and municipalities. Biennial follow-up surveys were conducted in 2013, 2015, 2018, and 2020. Trained researchers measured physical parameters such as waist circumference and height using standardized equipment, and participants also underwent blood tests. Detailed information about the study design has been previously published (18). All participants of CHARLS in the survey provided informed consent, and the study protocol received approval from the Biomedical Ethics Review Committee of Peking University (IRB00001052-11015).

### 2.2 Study design and population

**Figure 1:**
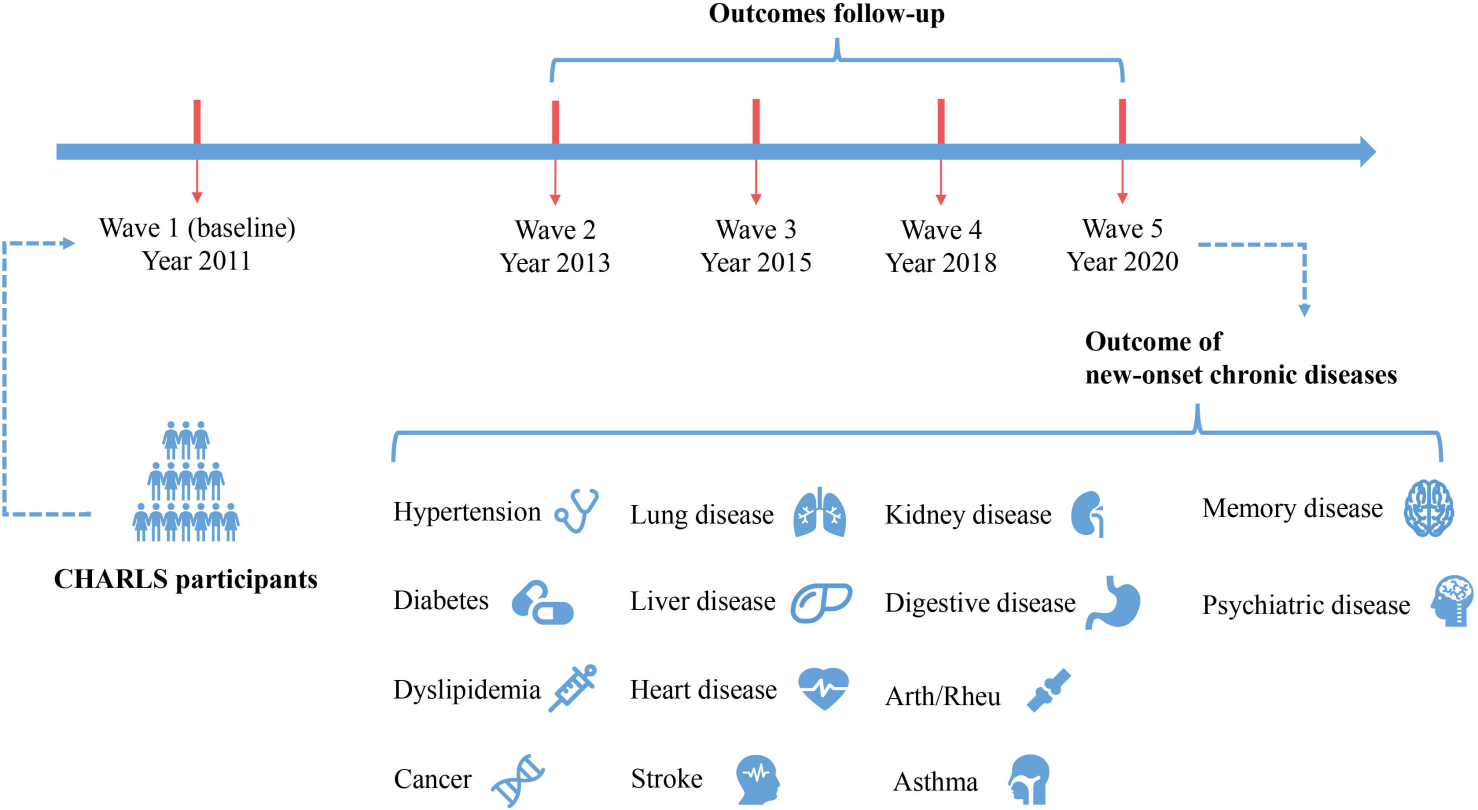
Study design and conceptual drawings. Arth/Rheu, arthritis or rheumatism; CHARLS, China Health and Retirement Longitudinal Study.

**Figure 2:**
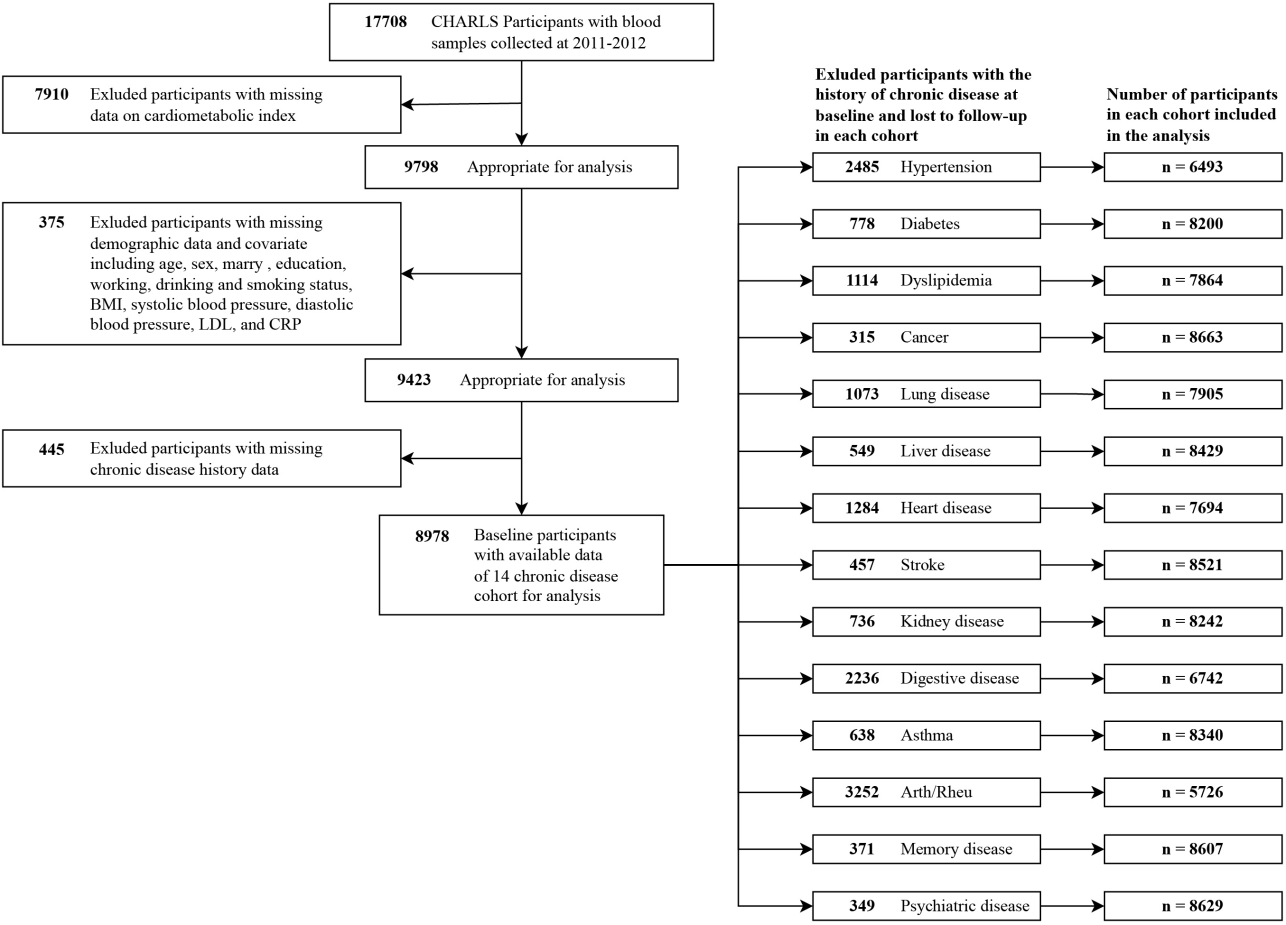
Flow diagram of the screening of the CHARLS participants. Arth/Rheu, arthritis or rheumatism; BMI, body mass index; CHARLS, China Health and Retirement Longitudinal Study; LDL, low density lipoprotein; CRP, C-reactive protein.

**Figure 1** depicts the study design of this investigation. Beginning with 2011 data as the baseline, we conducted follow-ups for 14 types of chronic disease outcomes in 2013, 2015, 2018, and 2020, resulting in the creation of separate cohorts. The study population and inclusion criteria are outlined in **Figure 2**. Individuals with missing CMI data (n=7910), absent demographic and covariate data (n=375), and lacking chronic disease history data (n=445) were excluded at baseline. Subsequently, individuals with a history of the corresponding chronic disease at baseline were also excluded, as well as those lost to follow-up in each chronic disease cohort.

### 2.3 Assessment of chronic disease

During the follow-up period of each chronic disease cohort, participants who responded affirmatively to the question, “Have you been diagnosed with…?” by a doctor were defined as having experienced a new-onset chronic disease and were considered as reaching an endpoint. Time to onset was defined as the midpoint between the onset wave follow-up and the last participant follow-up (19).

### 2.4 Diagnosis of CMI

CMI was calculated according to the following formula: CMI = TG (mmol/L)/HDL− C (mmol/L)×WHtR, where WHtR = waist circumference (cm)/height (cm) (7).

### 2.5 Covariates

Informed by published research and clinical judgment, several variables were identified as potential confounders, including age, sex, marital status, education, employment status, alcohol consumption, smoking status, body mass index (BMI), systolic blood pressure, diastolic blood pressure, low-density lipoprotein (LDL), and C-reactive protein (CRP) (20–22). Additionally, in line with previous studies and acknowledging the interconnectedness among chronic diseases, we incorporated the history of the 14 chronic diseases at baseline as a covariate (excluding the specific chronic disease under investigation in each cohort) across the disease cohorts to adjust for the influence of baseline history on the outcome event (21). Marital status was categorized as married and others; education was categorized as less than high school, high school and vocational training, and college and above; working status was categorized as no and yes; while drinking and smoking status were categorized as never, former, and now.

### 2.6 Statistical analysis

The baseline characteristics were delineated with respect to CMI quartiles. Continuous variables were presented as means and standard deviation (SD), while categorical variables were described using numerical counts and percentage frequencies (%). For continuous variables, between-group comparisons were made using the independent samples t-test, and one-way analysis of variance (ANOVA) was used to compare differences between groups. The chi-square test was used to assess differences between categorical variables and the Wilcoxon rank sum test was used to assess differences between ordered categorical variables.

Cox proportional hazards models were employed to assess the association between CMI and new-onset chronic diseases. Model 1, the crude model, did not incorporate any covariates. Model 2 was adjusted for age, sex, marital status, education, employment status, alcohol consumption, smoking status, BMI, systolic blood pressure, diastolic blood pressure, LDL, CRP, and the history of 14 chronic diseases at baseline (excluding the specific chronic disease under investigation in each cohort). Categorical variables were treated as continuous variables to check for potential linear trends in the model. Following adjustment for all potential confounders, potential nonlinear effects were addressed using restricted cubic spline (RCS) models with 3 knots positioned at 10%, 50%, and 90%. Kaplan-Meier survival curves were used to estimate survival in quartiles of baseline CMI for each cohort, and comparisons were made using the log-rank test.

Subgroup analyses were performed based on sex, marital status, education level, alcohol consumption, smoking status. To reduce reverse causation bias, we reanalyzed the data after excluding participants who experienced outcome events during the first follow-up (wave 2). Additionally, to mitigate the impact of missing variables on the results, we addressed missing values through multiple imputation with 5 replications using a chained equation approach.

All analyses were performed using R (version 4.2.3) with the “survival” package, and Free Software Foundation statistics software (version 1.9.2). The “forestploter” package was employed for plotting forest plots, the “jskm” package for generating Kaplan-Meier survival curves, and the “mice” package for multiple imputation. Statistical significance was defined as 2-sided p-values <0.05.

## 3 Results

### 3.1 Characteristics of the participants

**Table 1** presents the baseline characteristics of participants stratified by CMI quartiles. The mean age of participants was 58.62 years (SD, 9.61), with 4,849 (54.01%) being female. Participants in higher quartiles were more likely to be female, employed, have higher BMI and LDL levels, while showing lower HDL levels compared to those in lower quartiles. Additionally, they had a higher incidence of chronic diseases (including hypertension, diabetes, hyperlipidemia, heart disease, and stroke).

**Table 1:** Baseline characteristics of the CHARLS participants by cardiometabolic index quartile.

Characteristics	Total N=8978	Quartile 1 N=2284	Quartile 2 N=2248	Quartile 3 N=2228	Quartile 4 N=2218	*P* value
Age, mean (SD), years	58.62 (9.61)	59.02 (9.84)	58.67 (9.93)	58.53 (9.53)	58.24 (9.09)	0.160
Sex, No (%)						<0.001
Female	4849 (54.01)	1050 (45.97)	1191 (52.98)	1297 (58.21)	1311 (59.11)	
Male	4129 (45.99)	1234 (54.03)	1057 (47.02)	931 (41.79)	907 (40.89)	
Marry, No (%)						0.088
Married	7542 (84.01)	1908 (83.54)	1870 (83.19)	1863 (83.62)	1901 (85.71)	
Others	1436 (15.99)	376 (16.46)	378 (16.81)	365 (16.38)	317 (14.29)	
Education level, No (%)						0.028
Less than high school	8114 (90.38)	2085 (91.29)	2033 (90.44)	2009 (90.17)	1987 (89.59)	
High school and vocational training	760 (8.47)	184 (8.06)	190 (8.45)	195 (8.75)	191 (8.61)	
College and above	104 (1.16)	15 (0.66)	25 (1.11)	24 (1.08)	40 (1.80)	
Working status, No (%)						<0.001
No	3042 (33.88)	631 (27.63)	730 (32.47)	778 (34.92)	903 (40.71)	
Yes	5936 (66.12)	1653 (72.37)	1518 (67.53)	1450 (65.08)	1315 (59.29)	
Drinking status, No (%)						<0.001
Never	5502 (61.28)	1231 (53.90)	1377 (61.25)	1443 (64.77)	1451 (65.42)	
Former	750 (8.35)	165 (7.22)	197 (8.76)	215 (9.65)	173 (7.80)	
Now	2726 (30.36)	888 (38.88)	674 (29.98)	570 (25.58)	594 (26.78)	
Smoking status, No (%)						<0.001
Never	5491 (61.16)	1256 (54.99)	1355 (60.28)	1457 (65.39)	1423 (64.16)	
Former	792 (8.82)	200 (8.76)	182 (8.10)	188 (8.44)	222 (10.01)	
Now	2695 (30.02)	828 (36.25)	711 (31.63)	583 (26.17)	573 (25.83)	
BMI, mean (SD), kg/m2	24.00 (27.85)	21.53 (3.24)	22.78 (3.49)	24.18 (3.72)	27.59 (55.53)	<0.001
Systolic blood pressure, mean (SD), mmHg	129.29 (21.49)	125.91 (21.25)	127.07 (20.57)	130.85 (21.83)	133.46 (21.45)	<0.001
Diastolic blood pressure, mean (SD), mmHg	75.27 (12.26)	72.77 (12.04)	74.06 (11.68)	76.43 (12.46)	77.90 (12.19)	<0.001
LDL, mean (SD), mg/dL	116.42 (35.04)	110.72 (29.75)	118.20 (32.96)	124.20 (34.29)	112.66 (40.81)	<0.001
CRP, mean (SD), mg/L	2.76 (7.76)	2.69 (8.76)	2.63 (6.94)	2.73 (7.82)	3.00 (7.39)	<0.001
History of chronic disease = yes						
Hypertension, No (%)	3677 (40.96)	717 (31.39)	769 (34.21)	1009 (45.29)	1182 (53.29)	<0.001
Diabetes, No (%)	1523 (16.96)	218 (9.54)	278 (12.37)	367 (16.47)	660 (29.76)	<0.001
Dyslipidemia, No (%)	894 (9.96)	107 (4.68)	165 (7.34)	220 (9.87)	402 (18.12)	<0.001
Cancer, No (%)	78 (0.87)	11 (0.48)	21 (0.93)	19 (0.85)	27 (1.22)	0.065
Lung disease, No (%)	872 (9.71)	256 (11.21)	236 (10.50)	199 (8.93)	181 (8.16)	0.002
Liver disease, No (%)	311 (3.46)	85 (3.72)	78 (3.47)	70 (3.14)	78 (3.52)	0.762
Heart disease, No (%)	1075 (11.97)	210 (9.19)	229 (10.19)	278 (12.48)	358 (16.14)	<0.001
Stroke, No (%)	222 (2.47)	42 (1.84)	47 (2.09)	55 (2.47)	78 (3.52)	0.002
Kidney disease, No (%)	876 (9.76)	222 (9.72)	213 (9.48)	217 (9.74)	224 (10.10)	0.918
Digestive disease, No (%)	2045 (22.78)	571 (25.00)	528 (23.49)	481 (21.59)	465 (20.96)	0.005
Arth/Rheu, No (%)	3088 (34.40)	750 (32.84)	775 (34.48)	797 (35.77)	766 (34.54)	0.226
Asthma, No (%)	408 (4.54)	118 (5.17)	103 (4.58)	103 (4.62)	84 (3.79)	0.171
Memory disease, No (%)	133 (1.48)	33 (1.44)	36 (1.60)	26 (1.17)	38 (1.71)	0.463
Psychiatric disease, No (%)	112 (1.25)	35 (1.53)	27 (1.20)	24 (1.08)	26 (1.17)	0.538
TG, mean (SD), mg/dL	131.45 (95.51)	64.21 (18.66)	92.28 (23.05)	127.55 (29.72)	244.30 (127.22)	<0.001
HDL, mean (SD), mg/dL	51.21 (15.25)	66.39 (14.52)	54.07 (10.58)	46.82 (8.68)	37.09 (8.43)	<0.001

Arth/Rheu, arthritis or rheumatism; BMI, body mass index; CHARLS, China Health and Retirement Longitudinal Study; CRP, C-reactive protein; LDL, low density lipoprotein; SD, standard deviation; TG, triglycerides.

### 3.2 Association between CMI and new-onset chronic diseases

**Figure 3:**
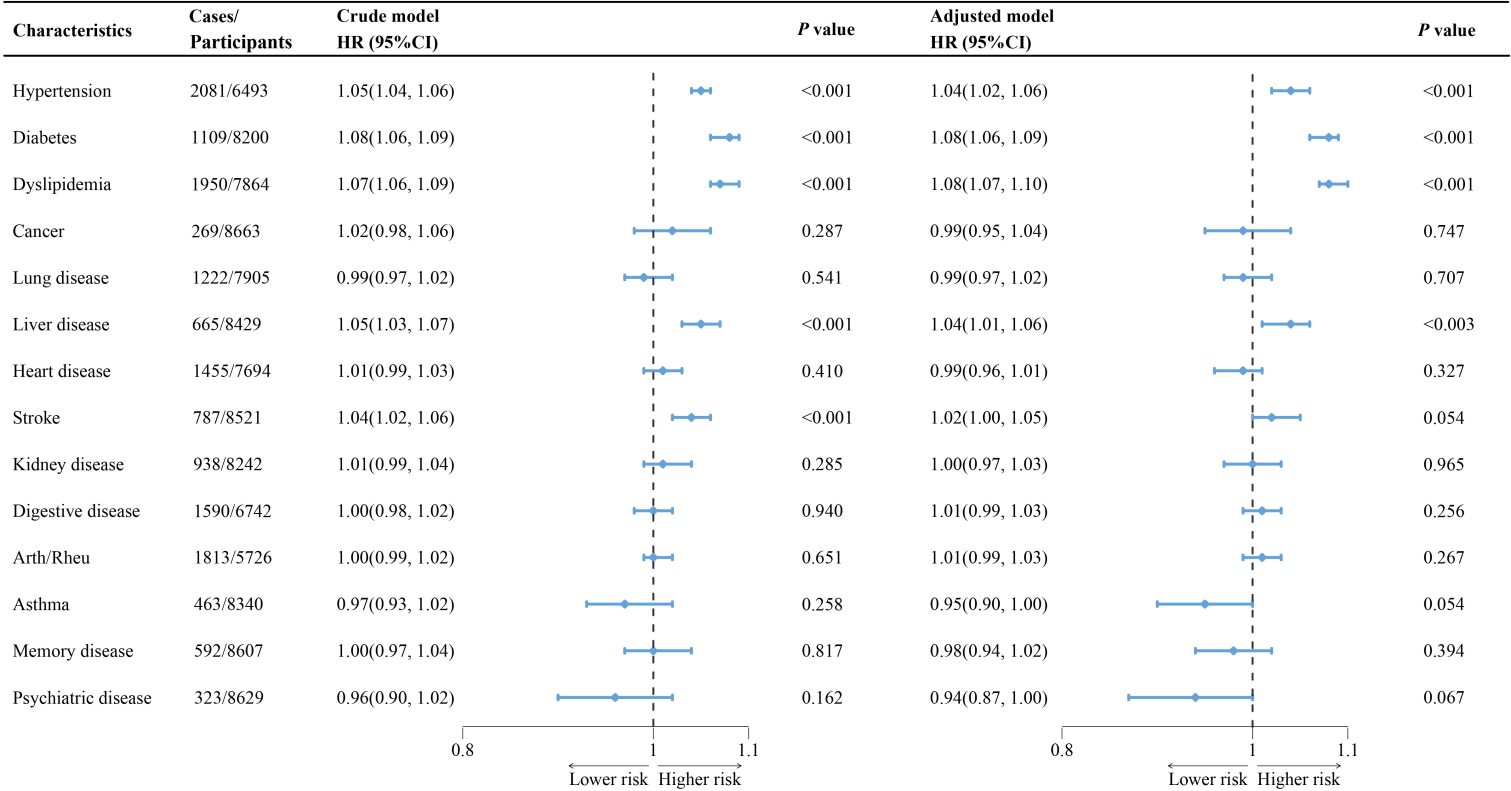
Association of cardiometabolic index with new-onset chronic diseases of the CHARLS participants. The crude models were not adjusted for any covariates, while adjusted models were adjusted for age, sex, marry, education, working, drinking, smoking, BMI, systolic blood pressure, diastolic blood pressure, LDL, CRP, and the history of 14 chronic diseases at baseline (excluding the specific chronic disease under investigation in each cohort). Arth/Rheu, arthritis or rheumatism; BMI, body mass index; CHARLS, China Health and Retirement Longitudinal Study; CI, confidence interval; HR, hazard ratio; LDL, low density lipoprotein; CRP, C-reactive protein.

As shown in **Figure 3**, higher CMI levels were associated with an increased risk of new-onset hypertension (HR=1.05, 95% CI=1.04-1.06, *P*<0.001), diabetes (HR=1.08, 95% CI=1.06-1.09, *P*<0.001), dyslipidemia (HR=1.07, 95% CI=1.06-1.09, *P*<0.001), liver disease (HR=1.05, 95% CI=1.03-1.07, *P*<0.003), and stroke (HR=1.04, 95% CI=1.02-1.06, *P*<0.001). In the fully adjusted model, these associations remained significant, except for the association between CMI and the risk of new-onset stroke (HR=1.02, 95% CI=1.00-1.05, *P*=0.054). Those results above remained robust after multiple imputation (**Supplementary Table 1**).

**Figure 4:**
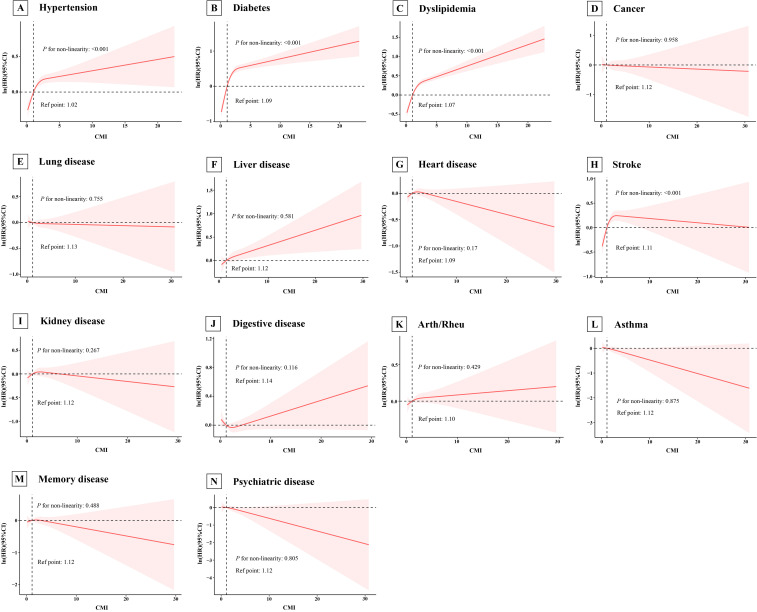
Association of cardiometabolic index with new-onset chronic diseases of the CHARLS participants by RCS. The model adjusted for age, sex, marry, education, working, drinking, smoking, BMI, systolic blood pressure, diastolic blood pressure, LDL, CRP, and the history of 14 chronic diseases at baseline (excluding the specific chronic disease under investigation in each cohort). Arth/Rheu, arthritis or rheumatism; CHARLS, China Health and Retirement Longitudinal Study; CMI, cardiometabolic index; LDL, low density lipoprotein; CRP, C-reactive protein; RCS, restricted cubic spline. **(A)** Hypertension; **(B)** Diabetes; **(C)** Dyslipidemia; **(D)** Cancer; **(E)** Lung disease; **(F)** Liver disease; **(G)** Heart disease; **(H)** Stroke; **(I)** Kidney disease; **(J)** Digestive disease; **(K)** Arth/Rheu; **(L)** Asthma; **(M)** Memory disease; **(N)** Psychiatric disease.

Additionally, as presented in the **Supplementary Table 2**, in the fully adjusted model, participants in the highest CMI quartile had a significantly higher risk of developing the aforementioned chronic diseases compared to those in the lowest quartile (hypertension: HR=1.02, 95% CI=1.00-1.05, *P*=0.054; diabetes: HR=1.02, 95% CI=1.00-1.05, *P*=0.054; dyslipidemia: HR=1.02, 95% CI=1.00-1.05, *P*=0.054; liver disease: HR=1.02, 95% CI=1.00-1.05, *P*=0.054; stroke: HR=1.02, 95% CI=1.00-1.05, *P*=0.054), with significant trend test (all *P* for trend<0.001), and the results remained robust after multiple imputation (**Supplementary Table 3**). Moreover, RCS analysis indicated significant nonlinear associations between CMI and the risk of the above new-onset chronic diseases (**Figure 4**, all *P* for non-linearity<0.001).

### 3.3 Additional and subgroup analyses

**Figure 5:**
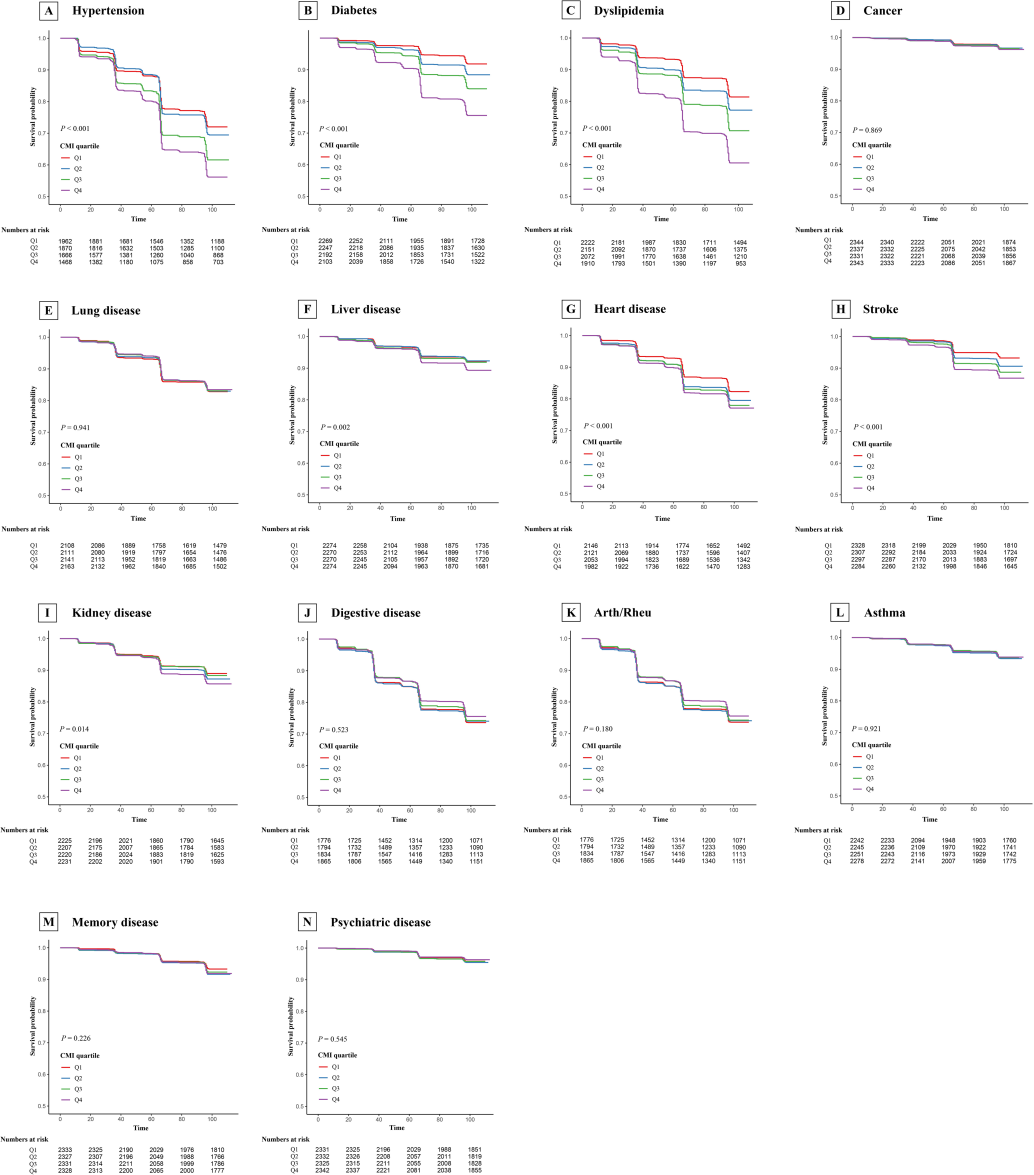
Kaplan-Meier survival curve for new-onset chronic diseases of CHARLS participants by cardiometabolic index quartile. Arth/Rheu, arthritis or rheumatism; CHARLS, China Health and Retirement Longitudinal Study, CMI, cardiometabolic index. **(A)** Hypertension; **(B)** Diabetes; **(C)** Dyslipidemia; **(D)** Cancer; **(E)** Lung disease; **(F)** Liver disease; **(G)** Heart disease; **(H)** Stroke; **(I)** Kidney disease; **(J)** Digestive disease; **(K)** Arth/Rheu; **(L)** Asthma; **(M)** Memory disease; **(N)** Psychiatric disease.

**Figure 5** displays the Kaplan-Meier survival curves for new-onset chronic diseases stratified by CMI quartile. Subgroup analyses found significant interactions between working status and new-onset diabetes, as well as smoking status and new-onset diabetes and stroke, with no significant interactions found for the remaining subgroups (**Supplementary Table 4**-**7**). Moreover, our results remained stable after excluding participants who experienced outcome events during the first follow-up (**Supplementary Table 8**).

## 4 Discussion

Higher levels of the CMI were associated with an increased risk of new-onset hypertension, diabetes, dyslipidemia, liver disease, and stroke, although the association with stroke was not significant after adjusting for all confounders. In the fully adjusted model, participants in the highest quartile of CMI had a significantly higher risk of these chronic diseases compared to those in the lowest quartile, with significant trend tests. Results remained robust after multiple imputations and exclude outcome events at the first follow-up. Moreover, RCS analyses showed a significant nonlinear relationship between CMI and the risk of the aforementioned new-onset chronic diseases. Interactions were found between working status and new-onset diabetes, as well as smoking and new-onset diabetes and stroke.

A cross-sectional study conducted in a Chinese community concluded that CMI was independently associated with a higher prevalence of hypertension (11). Meanwhile, another cross-sectional study based on a representative U.S. population found that CMI was significantly associated with the development of diabetes mellitus and hypertension combined with hyperuricemia (15, 23). Our findings, obtained from a longitudinal study of a representative sample, were consistent with these studies. However, we were unable to explore further the correlation between CMI and hyperuricemia due to the unavailability of blood test data from CHARLS after 2013.

A retrospective cohort study conducted in a Japanese population concluded that elevated baseline CMI levels were associated with diabetes (24). Additionally, insulin resistance and metabolic syndrome have been demonstrated to be strongly associated with diabetes (25, 26). In Chinese patients with diabetes, elevated CMI was significantly associated with insulin resistance (27). An Italian population-based study also found that CMI demonstrated excellent predictive efficacy for metabolic syndrome in adult obese patients (28). These findings align with our results. However, our study found that the association between CMI and stroke was not significant after adjusting for all confounders. In contrast, both population-based cross-sectional studies revealed a positive association between CMI and stroke risk (14, 29). Further prospective studies are needed to clarify this relationship in the future.

Cao et al. developed a machine learning model based on a longitudinal study and concluded that CMI would impact non-alcoholic fatty liver disease (NAFLD) incidence (30). Meanwhile, Duan et al. found that CMI was positively associated with the incidence of MAFLD (12). Our study also demonstrated a significant association between CMI and the risk of new-onset chronic liver disease. Additionally, although our study did not find an association between CMI and chronic digestive diseases, a retrospective study concluded that CMI predicts the severity of hyperlipidemic acute pancreatitis (31), suggesting its potential utility in acute diseases of the digestive system.

Increased CMI reflects higher levels of obesity and lipids, which lead to chronic systemic inflammation and metabolic syndrome, playing a crucial role in the development and progression of chronic diseases (32, 33). Specifically, obesity leads to increased intestinal permeability and lipids, while elevated levels of circulating gut bacteria and free fatty acids may promote pro-inflammatory macrophage infiltration by binding to pattern recognition receptors such as TLR4 and TLR2. This process also involves the release of chemokines and pro-inflammatory cytokines (e.g., tumor necrosis factor (TNF)-α, interleukin (IL)-1β, and IL-6), which contribute to and promote systemic inflammation and metabolic syndrome (34, 35). Systemic inflammation and metabolic syndrome are characteristic of many common chronic diseases (36, 37).

To our knowledge, this is the first study to examine the association between CMI and new-onset chronic diseases in a national prospective longitudinal study, yielding results of relatively high quality. The rigorous quality control procedures implemented by CHARLS in data collection enabled us to evaluate this association in a sizable and diverse sample of adults in China. More importantly, compared with studying only a single disease, the inclusion of baseline history of 14 chronic diseases as covariates (excluding the specific chronic disease under investigation in each cohort) across disease cohorts minimized the influence of different chronic disease histories at baseline on outcome events.

However, this study has some limitations. Firstly, the diagnosis of chronic conditions and some covariates relied on self-report, potentially introducing recall bias. Employing more objective and scientifically robust assessment tools or modalities could enhance the reliability of the data and outcomes. Secondly, the possibility of confounding effects from residual factors related to unconsidered chronic conditions, measurement error, and unknown confounders cannot be entirely excluded. Thirdly, this study could not assess the dynamic changes in CMI or its association with outcomes due to data structure limitations. Future research should include more frequent CMI measurements during follow-up to explore these dynamics. Finally, the generalizability of the study’s results is limited to China, and further prospective studies and intervention trials are needed in other countries and regions in the future.

## 5 Conclusions

In this national prospective longitudinal study, we found a significant positive association between CMI and the risk of new-onset chronic diseases such as hypertension, diabetes, dyslipidemia, and liver disease. In a future where the burden and impact of chronic disease are increasing, CMI is expected to serve as an effective marker for early identification of chronic disease risk.

## Data Availability

Publicly available datasets were analyzed in this study. This data can be found here: The China Health and Retirement Longitudinal Study can be publicly accessed at https://charls.pku.edu.cn/.
