# Supplementary material for: Cardiometabolic index and the risk of new-onset chronic diseases: results of a national prospective longitudinal study
Source: Front Endocrinol (Lausanne). 2024 Oct 21;15:1446276. doi: 10.3389/fendo.2024.1446276 (PMC11532088; doi:10.3389/fendo.2024.1446276)
Supplement: Supplementary file 1 [file Table1.docx]

**Supplementary Materials Files**

To: **Cardiometabolic index and the risk of new-onset chronic diseases: Results of a national prospective longitudinal study**

by Liyuan Zhuo, Mingxi Lai, Lulu Wan, Xuan Zhang, Ronglin Chen

**Supplementary Table 1. Association of cardiometabolic index with new-onset chronic diseases of the CHARLS participants after multiple imputation.**

**Supplementary Table 2. Association of cardiometabolic index quartile with new-onset chronic diseases of the CHARLS participants.**

**Supplementary Table 3. Association of cardiometabolic index quartile with new-onset chronic diseases of the CHARLS participants after multiple imputation.**

**Supplementary Table 4. Association of cardiometabolic index with new-onset hypertension, diabetes, dyslipidemia, and cancer of the CHARLS participants in different subgroups.**

**Supplementary Table 5. Association of cardiometabolic index with new-onset lung disease, liver disease, heart disease, and stroke of the CHARLS participants in different subgroups.**

**Supplementary Table 6. Association of cardiometabolic index with new-onset kidney disease, digestive disease, arthritis, and asthma of the CHARLS participants in different subgroups.**

**Supplementary Table 7. Association of cardiometabolic index with new-onset memory disease and psychiatric disease of the CHARLS participants in different subgroups.**

**Supplementary Table 8.** **Association of cardiometabolic index with new-onset chronic diseases of the CHARLS participants after** **excluding participants who experienced outcome events during wave 2.**

**Supplementary Table 1. Association of cardiometabolic index with new-onset chronic diseases of the CHARLS participants after multiple imputation.**

| **Characteristics** | **Model 1^a^** | | **Model 2^b^** | |
| --- | --- | --- | --- | --- |
|  | **HR(95%CI)** | ***P* value^a^** | **HR(95%CI)** | ***P* value^a^** |
| Hypertension | 1.03(1.02, 1.03) | <0.001 | 1.04(1.02, 1.06) | <0.001 |
| Diabetes | 1.03(1.02, 1.04) | <0.001 | 1.08(1.07, 1.10) | <0.001 |
| Dyslipidemia | 1.03(1.02, 1.04) | <0.001 | 1.08(1.07, 1.10) | <0.001 |
| Cancer | 1.00(0.97, 1.04) | 0.838 | 0.99(0.95, 1.04) | 0.747 |
| Lung disease | 1.00(0.99, 1.02) | 0.774 | 1.00(0.97, 1.02) | 0.707 |
| Liver disease | 1.03(1.02, 1.04) | <0.001 | 1.04(1.01, 1.06) | 0.003 |
| Heart disease | 1.01(0.99, 1.02) | 0.341 | 0.99(0.96, 1.01) | 0.327 |
| Stroke | 1.02(1.00, 1.03) | 0.010 | 1.02(1.00, 1.05) | 0.054 |
| Kidney disease | 1.01(0.99, 1.02) | 0.492 | 1.00(0.97, 1.03) | 0.934 |
| Digestive disease | 1.00(0.99, 1.02) | 0.844 | 1.01(0.99, 1.03) | 0.256 |
| Arth/Rheu | 1.01(1.00, 1.02) | 0.120 | 1.01(0.99, 1.03) | 0.267 |
| Asthma | 1.02(1.00, 1.04) | 0.094 | 0.95(0.90, 1.00) | 0.055 |
| Memory disease | 0.99(0.97, 1.02) | 0.663 | 0.98(0.94, 1.02) | 0.394 |
| Psychiatric disease | 0.97(0.92, 1.02) | 0.185 | 0.94(0.87, 1.01) | 0.067 |

**Abbreviations:** Arth/Rheu, arthritis or rheumatism; BMI, body mass index; CHARLS, China Health and Retirement Longitudinal Study; CI, confidence interval; HR, hazard ratio; LDL, low density lipoprotein; CRP, C-reactive protein.

^a^. Crude model.

^b^. Adjusted for age, sex, marry, education, working, drinking, smoking, BMI, systolic blood pressure, diastolic blood pressure, LDL, CRP, and the history of 14 chronic diseases at baseline (excluding the specific chronic disease under investigation in each cohort).

**Supplementary Table 2. Association of cardiometabolic index quartile with new-onset chronic diseases of the CHARLS participants.**

| **Characteristics** | **Crude model** | | **Adjusted model** | |
| --- | --- | --- | --- | --- |
|  | **HR(95%CI)** | ***P* value^a^** | **HR(95%CI)** | ***P* value^a^** |
| Hypertension |  |  |  |  |
| Q1 | Reference |  | Reference |  |
| Q2 | 1.11(0.98, 1.26) | 0.105 | 1.08(0.96, 1.23) | 0.210 |
| Q3 | 1.46(1.29, 1.65) | <0.001 | 1.26(1.11, 1.43) | <0.001 |
| Q4 | 1.75(1.55, 1.98) | <0.001 | 1.39(1.22, 1.58) | <0.001 |
| *P* for trend |  | <0.001 |  | <0.001 |
| Diabetes |  |  |  |  |
| Q1 | Reference |  | Reference |  |
| Q2 | 1.40(1.14, 1.71) | 0.001 | 1.32(1.08, 1.62) | 0.008 |
| Q3 | 1.95(1.61, 2.37) | <0.001 | 1.70(1.40, 2.06) | <0.001 |
| Q4 | 3.16(2.64, 3.79) | <0.001 | 2.67(2.22, 3.22) | <0.001 |
| *P* for trend |  | <0.001 |  | <0.001 |
| Dyslipidemia |  |  |  |  |
| Q1 | Reference |  | Reference |  |
| Q2 | 1.28(1.12, 1.48) | <0.001 | 1.17(1.02, 1.35) | 0.029 |
| Q3 | 1.67(1.46, 1.91) | <0.001 | 1.37(1.19, 1.57) | <0.001 |
| Q4 | 2.52(2.21, 2.86) | <0.001 | 2.07(1.81, 2.36) | <0.001 |
| *P* for trend |  | <0.001 |  | <0.001 |
| Cancer |  |  |  |  |
| Q1 | Reference |  | Reference |  |
| Q2 | 0.94(0.67, 1.33) | 0.737 | 0.92(0.65, 1.31) | 0.656 |
| Q3 | 0.95(0.67, 1.33) | 0.757 | 0.91(0.64, 1.29) | 0.591 |
| Q4 | 1.13(0.82, 1.57) | 0.456 | 0.95(0.67, 1.35) | 0.790 |
| *P* for trend |  | 0.466 |  | 0.782 |
| Lung disease |  |  |  |  |
| Q1 | Reference |  | Reference |  |
| Q2 | 0.94(0.80, 1.10) | 0.442 | 0.95(0.81, 1.11) | 0.531 |
| Q3 | 0.96(0.82, 1.13) | 0.643 | 0.99(0.84, 1.16) | 0.897 |
| Q4 | 0.92(0.78, 1.07) | 0.282 | 0.94(0.79, 1.11) | 0.453 |
| *P* for trend |  | 0.355 |  | 0.576 |
| Liver disease |  |  |  |  |
| Q1 | Reference |  | Reference |  |
| Q2 | 1.02(0.82, 1.28) | 0.857 | 1.02(0.81, 1.28) | 0.871 |
| Q3 | 1.05(0.84, 1.31) | 0.663 | 1.02(0.81, 1.28) | 0.897 |
| Q4 | 1.37(1.11, 1.69) | 0.003 | 1.21(0.96, 1.51) | 0.102 |
| *P* for trend |  | 0.003 |  | 0.116 |
| Heart disease |  |  |  |  |
| Q1 | Reference |  | Reference |  |
| Q2 | 1.17(1.01, 1.36) | 0.036 | 1.10(0.95, 1.28) | 0.209 |
| Q3 | 1.27(1.10, 1.47) | 0.002 | 1.08(0.93, 1.26) | 0.311 |
| Q4 | 1.33(1.15, 1.55) | <0.001 | 1.05(0.90, 1.23) | 0.512 |
| *P* for trend |  | <0.001 |  | 0.595 |
| Stroke |  |  |  |  |
| Q1 | Reference |  | Reference |  |
| Q2 | 1.43(1.14, 1.79) | 0.002 | 1.36(1.08, 1.70) | 0.008 |
| Q3 | 1.75(1.41, 2.17) | <0.001 | 1.51(1.21, 1.89) | <0.001 |
| Q4 | 2.09(1.69, 2.58) | <0.001 | 1.63(1.31, 2.04) | <0.001 |
| *P* for trend |  | <0.001 |  | <0.001 |
| Kidney disease |  |  |  |  |
| Q1 | Reference |  | Reference |  |
| Q2 | 1.15(0.96, 1.39) | 0.125 | 1.13(0.94, 1.36) | 0.199 |
| Q3 | 1.04(0.86, 1.26) | 0.664 | 1.01(0.83, 1.23) | 0.928 |
| Q4 | 1.27(1.06, 1.52) | 0.010 | 1.15(0.94, 1.40) | 0.181 |
| *P* for trend |  | 0.035 |  | 0.363 |
| Digestive disease |  |  |  |  |
| Q1 | Reference |  | Reference |  |
| Q2 | 0.97(0.84, 1.11) | 0.630 | 0.95(0.83, 1.10) | 0.501 |
| Q3 | 0.94(0.82, 1.08) | 0.384 | 0.94(0.81, 1.09) | 0.395 |
| Q4 | 0.89(0.77, 1.02) | 0.105 | 0.93(0.80, 1.09) | 0.379 |
| *P* for trend |  | 0.097 |  | 0.369 |
| Arth/Rheu |  |  |  |  |
| Q1 | Reference |  | Reference |  |
| Q2 | 1.09(0.96, 1.24) | 0.190 | 1.11(0.97, 1.27) | 0.115 |
| Q3 | 1.11(0.97, 1.26) | 0.116 | 1.14(0.99, 1.30) | 0.063 |
| Q4 | 1.00(0.88, 1.14) | 0.998 | 1.05(0.92, 1.21) | 0.463 |
| *P* for trend |  | 0.916 |  | 0.397 |
| Asthma |  |  |  |  |
| Q1 | Reference |  | Reference |  |
| Q2 | 1.09(0.85, 1.40) | 0.519 | 1.11(0.86, 1.43) | 0.414 |
| Q3 | 0.95(0.73, 1.23) | 0.701 | 1.03(0.79, 1.34) | 0.848 |
| Q4 | 0.91(0.70, 1.18) | 0.476 | 0.87(0.65, 1.15) | 0.323 |
| *P* for trend |  | 0.320 |  | 0.281 |
| Memory disease |  |  |  |  |
| Q1 | Reference |  | Reference |  |
| Q2 | 1.20(0.95, 1.51) | 0.129 | 1.18(0.94, 1.49) | 0.157 |
| Q3 | 1.10(0.87, 1.40) | 0.406 | 1.03(0.81, 1.31) | 0.817 |
| Q4 | 1.20(0.95, 1.51) | 0.129 | 1.00(0.78, 1.28) | 0.981 |
| *P* for trend |  | 0.225 |  | 0.684 |
| Psychiatric disease |  |  |  |  |
| Q1 | Reference |  | Reference |  |
| Q2 | 1.12(0.83, 1.51) | 0.460 | 1.11(0.82, 1.51) | 0.488 |
| Q3 | 1.04(0.76, 1.41) | 0.809 | 0.99(0.71, 1.36) | 0.932 |
| Q4 | 0.86(0.62, 1.19) | 0.358 | 0.77(0.54, 1.11) | 0.157 |
| *P* for trend |  | 0.318 |  | 0.138 |

The crude models were not adjusted for any covariates, while adjusted models were adjusted for age, sex, marry, education, working, drinking, smoking, BMI, systolic blood pressure, diastolic blood pressure, LDL, CRP, and the history of 14 chronic diseases at baseline (excluding the specific chronic disease under investigation in each cohort). **Abbreviations:** Arth/Rheu, arthritis or rheumatism; BMI, body mass index; CHARLS, China Health and Retirement Longitudinal Study; CI, confidence interval; HR, hazard ratio; LDL, low density lipoprotein; CRP, C-reactive protein.

**Supplementary Table 3. Association of cardiometabolic index quartile with new-onset chronic diseases of the CHARLS participants after** **multiple imputation.**

| **Characteristics** | **Crude model** | | **Adjusted model** | |
| --- | --- | --- | --- | --- |
|  | **HR(95%CI)** | ***P* value^a^** | **HR(95%CI)** | ***P* value^a^** |
| Hypertension |  |  |  |  |
| Q1 | Reference |  | Reference |  |
| Q2 | 1.09(0.96, 1.23) | 0.180 | 1.08(0.96, 1.23) | 0.212 |
| Q3 | 1.47(1.30, 1.65) | <0.001 | 1.26(1.11, 1.43) | <0.001 |
| Q4 | 1.75(1.55, 1.97) | <0.001 | 1.39(1.22, 1.58) | <0.001 |
| *P* for trend |  | <0.001 |  | <0.001 |
| Diabetes |  |  |  |  |
| Q1 | Reference |  | Reference |  |
| Q2 | 1.45(1.19, 1.77) | <0.001 | 1.33(1.08, 1.62) | 0.007 |
| Q3 | 2.06(1.70, 2.48) | <0.001 | 1.71(1.41, 2.08) | <0.001 |
| Q4 | 3.33(2.79, 3.98) | <0.001 | 2.71(2.25, 3.26) | <0.001 |
| *P* for trend |  | <0.001 |  | <0.001 |
| Dyslipidemia |  |  |  |  |
| Q1 | Reference |  | Reference |  |
| Q2 | 1.28(1.11, 1.47) | <0.001 | 1.17(1.02, 1.35) | 0.029 |
| Q3 | 1.70(1.49, 1.94) | <0.001 | 1.37(1.19, 1.57) | <0.001 |
| Q4 | 2.50(2.20, 2.84) | <0.001 | 2.07(1.81, 2.37) | <0.001 |
| *P* for trend |  | <0.001 |  | <0.001 |
| Cancer |  |  |  |  |
| Q1 | Reference |  | Reference |  |
| Q2 | 0.97(0.70, 1.35) | 0.864 | 0.93(0.66, 1.31) | 0.656 |
| Q3 | 1.02(0.73, 1.41) | 0.930 | 0.91(0.64, 1.29) | 0.591 |
| Q4 | 1.11(0.80, 1.53) | 0.535 | 0.95(0.67, 1.35) | 0.790 |
| *P* for trend |  | 0.497 |  | 0.782 |
| Lung disease |  |  |  |  |
| Q1 | Reference |  | Reference |  |
| Q2 | 0.99(0.85, 1.15) | 0.852 | 0.95(0.81, 1.11) | 0.531 |
| Q3 | 0.98(0.84, 1.14) | 0.754 | 0.99(0.84, 1.16) | 0.897 |
| Q4 | 0.95(0.82, 1.11) | 0.540 | 0.94(0.79, 1.11) | 0.453 |
| *P* for trend |  | 0.534 |  | 0.576 |
| Liver disease |  |  |  |  |
| Q1 | Reference |  | Reference |  |
| Q2 | 0.97(0.78, 1.20) | 0.755 | 1.02(0.81, 1.28) | 0.871 |
| Q3 | 1.03(0.84, 1.28) | 0.757 | 1.02(0.81, 1.28) | 0.897 |
| Q4 | 1.35(1.11, 1.66) | 0.003 | 1.21(0.96, 1.51) | 0.102 |
| *P* for trend |  | 0.002 |  | 0.116 |
| Heart disease |  |  |  |  |
| Q1 | Reference |  | Reference |  |
| Q2 | 1.19(1.03, 1.37) | 0.020 | 1.10(0.95, 1.28) | 0.209 |
| Q3 | 1.28(1.11, 1.48) | 0.001 | 1.08(0.93, 1.26) | 0.311 |
| Q4 | 1.35(1.17, 1.55) | <0.001 | 1.05(0.90, 1.23) | 0.512 |
| *P* for trend |  | <0.001 |  | 0.595 |
| Stroke |  |  |  |  |
| Q1 | Reference |  | Reference |  |
| Q2 | 1.40(1.12, 1.74) | 0.003 | 1.36(1.08, 1.70) | 0.008 |
| Q3 | 1.70(1.38, 2.10) | <0.001 | 1.51(1.21, 1.89) | <0.001 |
| Q4 | 2.03(1.65, 2.49) | <0.001 | 1.63(1.31, 2.04) | <0.001 |
| *P* for trend |  | <0.001 |  | <0.001 |
| Kidney disease |  |  |  |  |
| Q1 | Reference |  | Reference |  |
| Q2 | 1.16(0.97, 1.38) | 0.115 | 1.13(0.94, 1.36) | 0.198 |
| Q3 | 1.05(0.88, 1.27) | 0.570 | 1.01(0.83, 1.23) | 0.924 |
| Q4 | 1.30(1.10, 1.55) | 0.003 | 1.15(0.94, 1.40) | 0.179 |
| *P* for trend |  | 0.011 |  | 0.360 |
| Digestive disease |  |  |  |  |
| Q1 | Reference |  | Reference |  |
| Q2 | 0.99(0.87, 1.13) | 0.905 | 0.95(0.83, 1.10) | 0.501 |
| Q3 | 0.96(0.84, 1.10) | 0.551 | 0.94(0.81, 1.09) | 0.395 |
| Q4 | 0.91(0.80, 1.04) | 0.177 | 0.93(0.80, 1.09) | 0.379 |
| *P* for trend |  | 0.151 |  | 0.369 |
| Arth/Rheu |  |  |  |  |
| Q1 | Reference |  | Reference |  |
| Q2 | 1.10(0.97, 1.25) | 0.143 | 1.11(0.98, 1.27) | 0.115 |
| Q3 | 1.14(1.00, 1.29) | 0.043 | 1.14(0.99, 1.30) | 0.063 |
| Q4 | 1.04(0.92, 1.18) | 0.546 | 1.05(0.92, 1.21) | 0.463 |
| *P* for trend |  | 0.450 |  | 0.397 |
| Asthma |  |  |  |  |
| Q1 | Reference |  | Reference |  |
| Q2 | 1.07(0.84, 1.36) | 0.598 | 1.11(0.86, 1.43) | 0.414 |
| Q3 | 1.01(0.79, 1.29) | 0.945 | 1.03(0.79, 1.34) | 0.848 |
| Q4 | 0.99(0.77, 1.26) | 0.905 | 0.87(0.65, 1.15) | 0.323 |
| *P* for trend |  | 0.793 |  | 0.281 |
| Memory disease |  |  |  |  |
| Q1 | Reference |  | Reference |  |
| Q2 | 1.25(1.00, 1.56) | 0.050 | 1.18(0.94, 1.49) | 0.157 |
| Q3 | 1.14(0.91, 1.44) | 0.251 | 1.03(0.81, 1.31) | 0.817 |
| Q4 | 1.21(0.97, 1.51) | 0.098 | 1.00(0.78, 1.28) | 0.981 |
| *P* for trend |  | 0.201 |  | 0.684 |
| Psychiatric disease |  |  |  |  |
| Q1 | Reference |  | Reference |  |
| Q2 | 1.10(0.82, 1.47) | 0.528 | 1.11(0.82, 1.51) | 0.488 |
| Q3 | 1.03(0.77, 1.39) | 0.845 | 0.99(0.71, 1.36) | 0.932 |
| Q4 | 0.88(0.65, 1.20) | 0.423 | 0.77(0.54, 1.11) | 0.157 |
| *P* for trend |  | 0.376 |  | 0.138 |

The crude models were not adjusted for any covariates, while adjusted models were adjusted for age, sex, marry, education, working, drinking, smoking, BMI, systolic blood pressure, diastolic blood pressure, LDL, CRP, and the history of 14 chronic diseases at baseline (excluding the specific chronic disease under investigation in each cohort). **Abbreviations:** Arth/Rheu, arthritis or rheumatism; BMI, body mass index; CHARLS, China Health and Retirement Longitudinal Study; CI, confidence interval; HR, hazard ratio; LDL, low density lipoprotein; CRP, C-reactive protein.

**Supplementary Table 4. Association of cardiometabolic index with new-onset hypertension, diabetes, dyslipidemia, and cancer of the CHARLS participants in different subgroups.**

| **Characteristics** | **Hypertension** | | **Diabetes** | | **Dyslipidemia** | | **Cancer** | |
| --- | --- | --- | --- | --- | --- | --- | --- | --- |
|  | **HR(95%CI)** | ***P* value^a^** | **HR(95%CI)** | ***P* value^a^** | **HR(95%CI)** | ***P* value** | **HR(95%CI)** | ***P* value^a^** |
| Sex, No. (%) |  | 0.921 |  | 0.755 |  | 0.587 |  | 0.136 |
| Female | 1.04(1.01, 1.07) |  | 1.08(1.05, 1.10) |  | 1.08(1.06, 1.10) |  | 0.95(0.86, 1.05) |  |
| Male | 1.04(1.01, 1.06) |  | 1.09(1.06, 1.11) |  | 1.09(1.06, 1.11) |  | 1.02(0.97, 1.08) |  |
| Marry, No (%) |  | 0.147 |  | 0.799 |  | 0.301 |  | 0.592 |
| Married | 1.03(1.01, 1.05) |  | 1.07(1.06, 1.09) |  | 1.09(1.07, 1.10) |  | 1.00(0.95, 1.05) |  |
| Others | 1.05(1.02, 1.09) |  | 1.11(1.05, 1.16) |  | 1.05(1.01, 1.08) |  | 0.93(0.77, 1.12) |  |
| Education, No (%) |  | 0.779 |  | 0.666 |  | 0.667 |  | 0.803 |
| Less than high school | 1.04(1.02, 1.06) |  | 1.08(1.06, 1.09) |  | 1.08(1.07, 1.10) |  | 1.00(0.95, 1.05) |  |
| High school and vocational training | 1.01(0.96, 1.06) |  | 1.11(1.04, 1.19) |  | 1.10(1.04, 1.17) |  | 0.97(0.80, 1.16) |  |
| College and above | 1.23(0.95, 1.59) |  | 1.26(0.95, 1.67) |  | 1.41(0.95, 2.10) |  | NA^c^ |  |
| Working, No (%) |  | 0.676 |  | 0.012 |  | 0.682 |  | 0.673 |
| No | 1.05(1.02, 1.07) |  | 1.04(1.01, 1.07) |  | 1.08(1.05, 1.10) |  | 0.99(0.93, 1.06) |  |
| Yes | 1.04(1.02, 1.06) |  | 1.10(1.08, 1.12) |  | 1.09(1.07, 1.10) |  | 1.00(0.94, 1.07) |  |
| Drinking status, No (%) |  | 0.720 |  | 0.727 |  | 0.054 |  | 0.149 |
| Never | 1.03(1.01, 1.06) |  | 1.09(1.07, 1.11) |  | 1.10(1.08, 1.12) |  | 0.99(0.93, 1.06) |  |
| Former | 1.08(1.00, 1.18) |  | 1.13(1.04, 1.22) |  | 1.12(1.02, 1.23) |  | 1.13(0.98, 1.29) |  |
| Now | 1.04(1.02, 1.07) |  | 1.07(1.04, 1.09) |  | 1.07(1.04, 1.09) |  | 0.95(0.85, 1.06) |  |
| Smoking status, No (%) |  | 0.841 |  | 0.033 |  | 0.075 |  | 0.307 |
| Never | 1.04(1.02, 1.06) |  | 1.09(1.07, 1.11) |  | 1.08(1.06, 1.10) |  | 1.01(0.96, 1.06) |  |
| Former | 1.03(0.99, 1.07) |  | 1.03(0.99, 1.08) |  | 1.07(1.03, 1.11) |  | 1.02(0.88, 1.17) |  |
| Now | 1.03(0.99, 1.08) |  | 1.09(1.07, 1.12) |  | 1.12(1.09, 1.15) |  | 0.90(0.76, 1.08) |  |

**Abbreviations:** Arth/Rheu, arthritis or rheumatism; BMI, body mass index; CHARLS, China Health and Retirement Longitudinal Study; CI, confidence interval; HR, hazard ratio; LDL, low density lipoprotein; CRP, C-reactive protein.

^a^. P for interaction.

^b^. All models were adjusted for age, sex, marry, education, working, drinking, smoking, BMI, systolic blood pressure, diastolic blood pressure, LDL, CRP, and the history of 14 chronic diseases at baseline (excluding the specific chronic disease under investigation in each cohort).

^c^. Since the number of participants at this level is too little, HR or its CI cannot be calculated.

**Supplementary Table 5. Association of cardiometabolic index with new-onset lung disease, liver disease, heart disease, and stroke of the CHARLS participants in different subgroups.**

| **Characteristics** | **Lung disease** | | **Liver disease** | | **Heart disease** | | **Stroke** | |
| --- | --- | --- | --- | --- | --- | --- | --- | --- |
|  | **HR(95%CI)** | ***P* value^a^** | **HR(95%CI)** | ***P* value^a^** | **HR(95%CI)** | ***P* value** | **HR(95%CI)** | ***P* value^a^** |
| Sex, No. (%) |  | 0.188 |  | 0.497 |  | 0.64 |  | 0.321 |
| Female | 0.98(0.94, 1.02) |  | 1.02(0.99, 1.06) |  | 0.99(0.96, 1.03) |  | 1.03(1.00, 1.07) |  |
| Male | 1.02(0.98, 1.06) |  | 1.05(1.02, 1.09) |  | 0.98(0.95, 1.02) |  | 1.01(0.97, 1.05) |  |
| Marry, No (%) |  | 0.402 |  | 0.313 |  | 0.465 |  | 0.256 |
| Married | 1.00(0.97, 1.03) |  | 1.04(1.02, 1.07) |  | 0.99(0.96, 1.02) |  | 1.03(1.00, 1.06) |  |
| Others | 0.98(0.90, 1.06) |  | 1.01(0.93, 1.09) |  | 0.96(0.89, 1.03) |  | 0.97(0.89, 1.06) |  |
| Education, No (%) |  | 0.329 |  | 0.726 |  | 0.473 |  | 0.992 |
| Less than high school | 0.99(0.96, 1.02) |  | 1.03(1.01, 1.06) |  | 0.99(0.96, 1.01) |  | 1.02(1.00, 1.05) |  |
| High school and vocational training | 1.05(0.98, 1.12) |  | 1.15(1.04, 1.26) |  | 0.99(0.92, 1.07) |  | 1.06(0.96, 1.16) |  |
| College and above | NA^b^ |  | NA^b^ |  | 0.90(0.56, 1.44) |  | NA^b^ |  |
| Working, No (%) |  | 0.302 |  | 0.284 |  | 0.295 |  | 0.076 |
| No | 1.02(0.98, 1.05) |  | 1.01(0.97, 1.06) |  | 0.98(0.93, 1.02) |  | 1.00(0.96, 1.04) |  |
| Yes | 0.98(0.95, 1.02) |  | 1.05(1.02, 1.08) |  | 1.00(0.97, 1.03) |  | 1.05(1.01, 1.08) |  |
| Drinking status, No (%) |  | 0.236 |  | 0.774 |  | 0.137 |  | 0.443 |
| Never | 1.02(0.99, 1.06) |  | 1.05(1.02, 1.08) |  | 0.99(0.96, 1.02) |  | 1.03(1.00, 1.06) |  |
| Former | 0.99(0.89, 1.09) |  | 1.07(0.93, 1.23) |  | 1.09(1.00, 1.19) |  | 0.93(0.79, 1.09) |  |
| Now | 0.96(0.91, 1.01) |  | 1.00(0.96, 1.05) |  | 0.97(0.92, 1.01) |  | 1.03(0.99, 1.07) |  |
| Smoking status, No (%) |  | 0.854 |  | 0.964 |  | 0.666 |  | 0.034 |
| Never | 1.00(0.97, 1.04) |  | 1.04(1.01, 1.08) |  | 1.00(0.97, 1.03) |  | 1.03(0.99, 1.06) |  |
| Former | 1.00(0.94, 1.07) |  | 1.05(1.00, 1.10) |  | 0.95(0.87, 1.03) |  | 0.99(0.91, 1.07) |  |
| Now | 0.98(0.92, 1.03) |  | 1.02(0.97, 1.07) |  | 0.97(0.92, 1.03) |  | 1.05(1.01, 1.10) |  |

**Abbreviations:** Arth/Rheu, arthritis or rheumatism; BMI, body mass index; CHARLS, China Health and Retirement Longitudinal Study; CI, confidence interval; HR, hazard ratio; LDL, low density lipoprotein; CRP, C-reactive protein.

^a^. P for interaction.

^b^. All models were adjusted for age, sex, marry, education, working, drinking, smoking, BMI, systolic blood pressure, diastolic blood pressure, LDL, CRP, and the history of 14 chronic diseases at baseline (excluding the specific chronic disease under investigation in each cohort).

^c^. Since the number of participants at this level is too little, HR or its CI cannot be calculated.

**Supplementary Table 6. Association of cardiometabolic index with new-onset kidney disease, digestive disease, arthritis, and asthma of the CHARLS participants in different subgroups.**

| **Characteristics** | **Kidney disease** | | **Digestive disease** | | **Arth/Rheu** | | **Asthma** | |
| --- | --- | --- | --- | --- | --- | --- | --- | --- |
|  | **HR(95%CI)** | ***P* value^a^** | **HR(95%CI)** | ***P* value^a^** | **HR(95%CI)** | ***P* value** | **HR(95%CI)** | ***P* value^a^** |
| Sex, No. (%) |  | 0.323 |  | 0.138 |  | 0.312 |  | 0.885 |
| Female | 0.99(0.95, 1.03) |  | 1.00(0.96, 1.04) |  | 1.02(1.00, 1.05) |  | 0.95(0.88, 1.03) |  |
| Male | 1.02(0.97, 1.06) |  | 1.02(0.99, 1.05) |  | 1.00(0.97, 1.03) |  | 0.96(0.89, 1.02) |  |
| Marry, No (%) |  | 0.429 |  | 0.701 |  | 0.306 |  | 0.153 |
| Married | 0.99(0.96, 1.02) |  | 1.02(0.99, 1.04) |  | 1.01(0.99, 1.03) |  | 0.96(0.92, 1.02) |  |
| Others | 1.04(0.98, 1.10) |  | 1.00(0.95, 1.05) |  | 1.01(0.98, 1.05) |  | 0.87(0.73, 1.04) |  |
| Education, No (%) |  | 0.401 |  | 0.717 |  | 0.463 |  | 0.765 |
| Less than high school | 0.99(0.96, 1.02) |  | 1.01(0.99, 1.03) |  | 1.01(0.99, 1.03) |  | 0.95(0.90, 1.00) |  |
| High school and vocational training | 1.02(0.96, 1.08) |  | 1.05(0.98, 1.12) |  | 0.94(0.85, 1.04) |  | 1.00(0.86, 1.17) |  |
| College and above | 1.35(1.04, 1.77) |  | 1.05(0.79, 1.40) |  | NA^c^ |  | NA^c^ |  |
| Working, No (%) |  | 0.469 |  | 0.285 |  | 0.493 |  | 0.301 |
| No | 0.98(0.93, 1.03) |  | 1.03(1.00, 1.06) |  | 1.02(0.99, 1.05) |  | 0.91(0.83, 0.99) |  |
| Yes | 1.01(0.98, 1.05) |  | 1.00(0.97, 1.03) |  | 1.00(0.98, 1.03) |  | 0.97(0.91, 1.03) |  |
| Drinking status, No (%) |  | 0.680 |  | 0.098 |  | 0.897 |  | 0.187 |
| Never | 1.01(0.97, 1.05) |  | 1.01(0.99, 1.04) |  | 1.01(0.99, 1.04) |  | 0.95(0.90, 1.02) |  |
| Former | 1.06(0.94, 1.18) |  | 0.94(0.82, 1.07) |  | 1.01(0.89, 1.14) |  | 0.75(0.54, 1.04) |  |
| Now | 0.97(0.92, 1.02) |  | 1.02(0.99, 1.05) |  | 1.00(0.96, 1.03) |  | 0.98(0.90, 1.07) |  |
| Smoking status, No (%) |  | 0.489 |  | 0.053 |  | 0.287 |  | 0.483 |
| Never | 1.02(0.98, 1.05) |  | 1.03(1.00, 1.05) |  | 1.00(0.98, 1.03) |  | 0.95(0.89, 1.02) |  |
| Former | 0.96(0.88, 1.05) |  | 0.92(0.83, 1.02) |  | 1.01(0.95, 1.06) |  | 0.99(0.88, 1.11) |  |
| Now | 0.99(0.93, 1.05) |  | 1.01(0.96, 1.05) |  | 1.04(1.00, 1.08) |  | 0.92(0.82, 1.04) |  |

**Abbreviations:** Arth/Rheu, arthritis or rheumatism; BMI, body mass index; CHARLS, China Health and Retirement Longitudinal Study; CI, confidence interval; HR, hazard ratio; LDL, low density lipoprotein; CRP, C-reactive protein.

^a^. P for interaction.

^b^. All models were adjusted for age, sex, marry, education, working, drinking, smoking, BMI, systolic blood pressure, diastolic blood pressure, LDL, CRP, and the history of 14 chronic diseases at baseline (excluding the specific chronic disease under investigation in each cohort).

^c^. Since the number of participants at this level is too little, HR or its CI cannot be calculated.

**Supplementary Table 7. Association of cardiometabolic index with new-onset memory disease and psychiatric disease of the CHARLS participants in different subgroups.**

| **Characteristics** | **Memory disease** | | **Psychiatric disease** | |
| --- | --- | --- | --- | --- |
|  | **HR(95%CI)** | ***P* value^a^** | **HR(95%CI)** | ***P* value^a^** |
| Sex, No. (%) |  | 0.34 |  | 0.453 |
| Female | 1.00(0.95, 1.05) |  | 0.95(0.85, 1.06) |  |
| Male | 0.96(0.90, 1.03) |  | 0.93(0.84, 1.02) |  |
| Marry, No (%) |  | 0.067 |  | 0.165 |
| Married | 1.00(0.96, 1.04) |  | 0.95(0.88, 1.02) |  |
| Others | 0.89(0.76, 1.04) |  | 0.87(0.66, 1.14) |  |
| Education, No (%) |  | 0.433 |  | 0.395 |
| Less than high school | 0.98(0.93, 1.02) |  | 0.92(0.85, 0.99) |  |
| High school and vocational training | 0.98(0.89, 1.08) |  | 1.06(0.89, 1.26) |  |
| College and above | 0.26(0.10, 0.67) |  | 1.09(0.98, 1.22) |  |
| Working, No (%) |  | 0.096 |  | 0.182 |
| No | 0.93(0.86, 1.00) |  | 0.88(0.78, 1.00) |  |
| Yes | 1.03(0.98, 1.08) |  | 0.98(0.90, 1.07) |  |
| Drinking status, No (%) |  | 0.084 |  | 0.326 |
| Never | 0.98(0.93, 1.04) |  | 0.95(0.87, 1.03) |  |
| Former | 1.07(0.98, 1.18) |  | 0.98(0.82, 1.17) |  |
| Now | 0.95(0.87, 1.04) |  | 0.85(0.70, 1.03) |  |
| Smoking status, No (%) |  | 0.851 |  | 0.785 |
| Never | 0.98(0.92, 1.03) |  | 0.92(0.84, 1.01) |  |
| Former | 0.97(0.89, 1.06) |  | 0.92(0.78, 1.08) |  |
| Now | 0.98(0.90, 1.07) |  | 0.98(0.84, 1.15) |  |

**Abbreviations:** Arth/Rheu, arthritis or rheumatism; BMI, body mass index; CHARLS, China Health and Retirement Longitudinal Study; CI, confidence interval; HR, hazard ratio; LDL, low density lipoprotein; CRP, C-reactive protein.

^a^. P for interaction.

^b^. All models were adjusted for age, sex, marry, education, working, drinking, smoking, BMI, systolic blood pressure, diastolic blood pressure, LDL, CRP, and the history of 14 chronic diseases at baseline (excluding the specific chronic disease under investigation in each cohort).

**Supplementary Table 8. Association of cardiometabolic index with new-onset chronic diseases of the CHARLS participants after excluding participants who experienced outcome events during wave 2.**

| **Characteristics** | **Model 1^a^** | | **Model 2^b^** | |
| --- | --- | --- | --- | --- |
|  | **HR(95%CI)** | ***P* value^a^** | **HR(95%CI)** | ***P* value^a^** |
| Hypertension | 1.05(1.04, 1.07) | <0.001 | 1.04(1.02, 1.06) | <0.001 |
| Diabetes | 1.08(1.06, 1.09) | <0.001 | 1.08(1.06, 1.10) | <0.001 |
| Dyslipidemia | 1.07(1.06, 1.09) | <0.001 | 1.08(1.06, 1.10) | <0.001 |
| Cancer | 1.02(0.97, 1.06) | 0.457 | 0.99(0.94, 1.04) | 0.685 |
| Lung disease | 0.98(0.96, 1.01) | 0.234 | 0.98(0.95, 1.01) | 0.243 |
| Liver disease | 1.04(1.02, 1.07) | <0.001 | 1.03(1.01, 1.06) | 0.015 |
| Heart disease | 1.01(0.99, 1.03) | 0.492 | 0.98(0.96, 1.01) | 0.266 |
| Stroke | 1.04(1.02, 1.06) | <0.001 | 1.03(1.00, 1.05) | 0.058 |
| Kidney disease | 1.02(0.99, 1.04) | 0.240 | 1.00(0.97, 1.03) | 0.973 |
| Digestive disease | 1.00(0.98, 1.02) | 0.899 | 1.02(0.99, 1.04) | 0.158 |
| Arth/Rheu | 1.01(0.99, 1.03) | 0.468 | 1.01(0.99, 1.03) | 0.240 |
| Asthma | 0.98(0.93, 1.02) | 0.354 | 0.95(0.90, 1.00) | 0.059 |
| Memory disease | 1.01(0.97, 1.04) | 0.728 | 0.98(0.94, 1.03) | 0.461 |
| Psychiatric disease | 0.96(0.90, 1.03) | 0.238 | 0.94(0.88, 1.01) | 0.117 |

**Abbreviations:** Arth/Rheu, arthritis or rheumatism; BMI, body mass index; CHARLS, China Health and Retirement Longitudinal Study; CI, confidence interval; HR, hazard ratio; LDL, low density lipoprotein; CRP, C-reactive protein.

^a^. Crude model.

^b^. Adjusted for age, sex, marry, education, working, drinking, smoking, BMI, systolic blood pressure, diastolic blood pressure, LDL, CRP, and the history of 14 chronic diseases at baseline (excluding the specific chronic disease under investigation in each cohort).
